# Synbiotic Compositions of *Bacillus megaterium* and Polyunsaturated Fatty Acid Salt Enable Self-Sufficient Production of Specialized Pro-Resolving Mediators

**DOI:** 10.3390/nu14112265

**Published:** 2022-05-28

**Authors:** Bodo Speckmann, Jessica Kleinbölting, Friedemann Börner, Paul M. Jordan, Oliver Werz, Stefan Pelzer, Heike tom Dieck, Tanja Wagner, Christiane Schön

**Affiliations:** 1 Evonik Operations GmbH, Rodenbacher Chaussee 4, 63457 Hanau, Germany; bodo.speckmann@evonik.com (B.S.); jessica.kleinboelting@evonik.com (J.K.); stefan.pelzer@evonik.com (S.P.); heike.tom-dieck@evonik.com (H.t.D.); 2 Department of Pharmaceutical/Medicinal Chemistry, Institute of Pharmacy, Friedrich Schiller University Jena, Philosophenweg 14, 07743 Jena, Germany; friedemann.boerner@uni-jena.de (F.B.); paul.jordan@uni-jena.de (P.M.J.); oliver.werz@uni-jena.de (O.W.); 3 BioTeSys GmbH, Schelztorstraße 54-56, 73728 Esslingen, Germany; t.wagner@biotesys.de

**Keywords:** probiotic, CYP450BM3, dietary supplement, drug, inflammatory bowel diseases, irritable bowel syndrome, *n*-3 PUFA, SPM

## Abstract

Specialized pro-resolving mediators (SPM) have emerged as crucial lipid mediators that confer the inflammation-resolving effects of omega-3 polyunsaturated fatty acids (*n*-3 PUFA). Importantly, SPM biosynthesis is dysfunctional in various conditions, which may explain the inconclusive efficacy data from *n*-3 PUFA interventions. To overcome the limitations of conventional *n*-3 PUFA supplementation strategies, we devised a composition enabling the self-sufficient production of SPM in vivo. *Bacillus megaterium* strains were fed highly bioavailable *n*-3 PUFA, followed by metabololipidomics analysis and bioinformatic assessment of the microbial genomes. All 48 tested *Bacillus megaterium* strains fed with the *n*-3 PUFA formulation produced a broad range of SPM and precursors thereof in a strain-specific manner, which may be explained by the CYP102A1 gene polymorphisms that we detected. A pilot study was performed to test if a synbiotic *Bacillus megaterium*/*n*-3 PUFA formulation increases SPM levels in vivo. Supplementation with a synbiotic capsule product led to significantly increased plasma levels of hydroxy-eicosapentaenoic acids (5-HEPE, 15-HEPE, 18-HEPE) and hydroxy-docosahexaenoic acids (4-HDHA, 7-HDHA) as well as eicosapentaenoic acid (EPA) and docosahexaenoic acid (DHA) in healthy humans. To the best of our knowledge, we report here for the first time the development and in vivo application of a self-sufficient SPM-producing formulation. Further investigations are warranted to confirm and expand these findings, which may create a new class of *n*-3 PUFA interventions targeting inflammation resolution.

## 1. Introduction

*n*-3 Polyunsaturated fatty acids (*n*-3 PUFA) mainly comprise the long-chain fatty acids alpha-linolenic acid (ALA), eicosapentaenoic acid (EPA), and docosahexaenoic acid (DHA). While dietary ALA primarily derives from some plants (flaxseed, soybean, canola), dietary EPA and DHA are mainly of seafood origin (fatty fish, krill, algae). *n*-3 PUFA can exert a range of health benefits [1,2] and are ingested with the regular diet or through fortified foods, dietary supplements, or pharmaceutical products [3,4]. *n*-3 PUFA, in general, have anti-inflammatory, cardio- and neuroprotective effects [2,5]. Their modes of action involve e.g., direct scavenging of reactive oxygen species, alteration of cell membrane fluidity, which subsequently affects cellular signaling events, modulation of the activity of transcription factors such as peroxisome proliferator-activated receptor γ (PPARγ) and nuclear factor κB (NFκB) that orchestrate the biosynthesis of pro- and anti-inflammatory cytokines, and competitive exclusion of substrates that are converted to pro-inflammatory lipid mediators by cyclooxygenases (COX) and lipoxygenases (LOX). More recently, several oxygenation products of *n*-3 PUFA and *n*-6 PUFA (e.g., arachidonic acid, AA) have been identified and functionally characterized as crucial mediators of their beneficial health effects, in particular with respect to the amelioration of chronic inflammatory conditions [6,7]. These products include maresins (MaR), E- and D-series resolvins (RvE and RvD), protectins (PD), lipoxins (LX), and precursors thereof such as 18-hydroxy-eicosapentaenoic acid (18-HEPE), 17-hydroxy-docosahexaenoic acid (17-HDHA), and 17,18-epoxyeicosatetraenoic acid (17,18-EEQ), collectively referred to as specialized pro-resolving mediators (SPM). SPM act as potent agonists of active inflammation resolution, signaling via e.g., G-protein coupled receptors at nano- to picomolar concentrations. Biosynthesis of SPM involves LOX, COX-2, and cytochrome P450 monooxygenases (CYP450), and has been described in detail for eukaryotic cells, in particular for granulocytes, monocytes, and macrophages [8,9,10]. Pro-resolving M2-like macrophages can express all enzymes that are required for SPM biosynthesis; other cell types expressing only selected enzymes can do so together with complementing cells via transcellular metabolism. 5-LOX is found in myeloid cells, 12-LOX in the skin and epithelial cells, 15-LOX in dendritic and enteric glial cells [11], and COX-2 and CYP450 isoforms in epithelial cells. There is only limited information about PUFA oxygenation by microorganisms in gastrointestinal tracts, amidst the generally low occurrence of oxygen-consuming enzymes therein. COX and LOX appear to be absent from gastrointestinal bacteria and archaea, with the exception of a 15-LOX expressed by pathogenic *Pseudomonas aeruginosa* [12]. CYP450s have been detected in a few genera within the phylum *Firmicutes*, with a particularly abundant presence in the genus *Bacillus* [13,14]. CYP102A1, also named CYP450BM3, is a bifunctional enzyme found in the species *Bacillus megaterium* that catalyzes the nicotinamide adenine dinucleotide phosphate (NADPH)-dependent hydroxylation of PUFA via consecutive oxygenase and reductase activities. For instance, purified CYP450BM3 derived from an expression vector construct has been shown to generate 18-HEPE from EPA in a cell-free reaction [15].

The effectiveness of SPM against a multitude of infectious and inflammatory diseases has been demonstrated in studies with rodents [6,9]. For example, RvE1, RvD2, PD1, and LXA_4_ enhance the clearance of pathogenic *Pseudomonas gingivalis*, *E. coli*, *Herpes simples*, Candida, H5N1 Influenza [16,17,18,19,20]. LXA_4_, LXB_4_, RvE1, RvE3, RvD1–5, RvD2, PD1, MaR1 and MaR2 are protective in animal and cellular models of Alzheimer’s disease [21], atherosclerosis [22], glaucoma [23], colitis [24], asthma [25], insulin resistance [26], arthritis [27], and pain [28]. Moreover, several precursors of SPM have themselves been shown to exert pro-resolving effects. For example, 18-HEPE counteracts the development of cardiovascular diseases by inhibiting monocyte adhesion to vascular endothelial cells [29] and by inhibiting pressure overload-induced maladaptive cardiac remodeling [30]. Similarly, 17,18-EEQ has cardio-protective, anti-arrhythmic, vasodilatory, and anti-inflammatory properties [5]. Paracrine secretion of AA-derived 15-hydroxyeicosatetraenoic acid (15-HETE) by enteric glial cells supports gut barrier function, a process that is impaired in e.g., Crohn’s disease [11].

Translation of these promising preclinical findings towards improving human health has, however, been challenging. Direct delivery of SPM by intravenous or intraperitoneal injection, as has been done in experimental studies, is not feasible for humans, particularly not in the context of preventive approaches. Oral delivery of SPM or SPM precursor-containing supplements or foods is not reasonable because of their relatively short half-life in biological fluids, which are therefore unlikely to reach their target tissue. Recent meta-analyses of *n*-3 PUFA/fish oil supplementation trials have yielded inconclusive or null results, especially with respect to the prevention of cardiovascular diseases [31], and also extending to inflammatory bowel diseases, asthma, and traits of the metabolic syndrome [2]. This lack of benefit contrasts with the effective treatment of the respective animal disease models by SPM [6]. We reason that the conversion of *n*-3 PUFA (and *n*-6 PUFA) to SPM is a prerequisite for delivering successful outcomes from any interventions aiming to prevent, cure, or treat inflammatory diseases with *n*-3 PUFA. We also conceive that the SPM-producing machinery is dysfunctional under certain conditions. These ideas are supported by findings of reduced (local or circulating) SPM levels in diabetic wounds [32], metabolic syndrome [33], asthma [34], ulcerative colitis [35], and Crohn’s disease [11,36], as well as reduced expression or activity of SPM-producing enzymes in, e.g., inflammatory bowel diseases (IBD) [11,37] and cystic fibrosis [38].

Here, we aimed at developing a formulation that can leverage the SPM-mediated benefits of *n*-3 PUFA intake irrespective of the various intrinsic (e.g., genetics, disease state, sex) and extrinsic (e.g., diet) determinants of the body’s SPM production. Therefore, we report a synbiotic approach based on the discovery of potent SPM-producing *Bacillus megaterium* strains using a fish oil-derived *n*-3 PUFA rich lysine salt as a substrate. Proof-of-principle that such an approach is a tool for increasing SPM levels in humans is also provided by testing a nutritional product composed of *Bacillus megaterium* DSM (Number of German Collection of Microorganisms and Cell Cultures) 32963 and an *n*-3 PUFA rich lysine salt in a 4-week pilot intervention trial.

## 2. Materials and Methods

### 2.1. Bacterial Cell Culture

*Bacillus megaterium* strains were precultured separately in a 10 mL Luria Bertami (LB) medium with 0.1% glucose (LBG) for 24 h at 30 °C and 200 rpm in a 100 mL flask. The complete culture was transferred to a 200 mL main culture in LBG. The main culture was grown for 6 h at 30 °C and 200 rpm in a 2 L flask. The cell culture was then harvested in 10 mL portions, the supernatant removed by centrifugation (4000 rpm, 15 min, room temperature) and the cell pellet resuspended in 10 mL LBG and 2 mL of liposomal *n*-3 PUFA stock solution containing 2.04 g/L of EPA. These cultures were incubated in 100 mL shaking flasks for 16 h at 30 °C and 200 rpm. Cell-free control reactions were performed in parallel to correct for spontaneously formed or background levels of *n*-3 PUFA metabolites. Supernatants were collected and subjected to metabololipidomics analysis of *n*-3 PUFA oxygenation products.

#### Preparation of Liposomal *n*-3 PUFA

The liposomal *n*-3 PUFA stock solution was prepared as follows: 0.8 g of dioleylphosphatidylcholine (DOPC, Lipoid GmbH, Ludwigshafen, Germany) were dissolved in 5 mL ethanol. Thereafter, 0.2 g of a lysine salt of fish oil-derived free *n*-3 PUFA (AvailOm^®^) were added and dissolved. The *n*-3 PUFA lysine salt contains around 33% of L-lysine and 67% of *n*-3 PUFA (30–35% EPA; 15–20% DHA; 11–25% other fatty acids including docosapentaenoic acid and the omega-6 fatty acids arachidonic acid, docosatetraenoic acid and docosaenoic acid isomer). A total of 1 mL of the respective solution was added dropwise to 20 mL of a 0.1 M phosphate buffer, pH = 8, at a temperature of 45 °C under intense stirring. Afterwards the dispersion was put on ice and sonified for 15 min to generate nanometer scale dispersions, presumably liposomes (Branson Sonifier, 100% amplitude, 50% impulse). Finally, the dispersion was sterile filtered through 0.2 µm syringe filters, and 6 mL of it was added to 4 mL of PBS to yield the liposomal *n*-3 PUFA stock solution that was used in the bacterial cell cultivations.

### 2.2. Lipid Mediator Metabololipidomics by UPLC-MS-MS

Lipid mediator (LM) analysis using ultra performance liquid chromatography-tandem mass spectrometer (UPLC-MS/MS) was performed as described previously [8] with some minor modifications [39]. Briefly, freshly thawed human plasma or *Bacillus megaterium* cultures were first mixed with the same volume of ice-cold methanol containing deuterium-labeled internal standards (200 nM d8–5S-HETE, d4-LTB4, d5-LXA4, d5-RvD2, d4-PGE2 and 10 µM d8-AA; Cayman Chemical/Biomol GmbH, Hamburg, Germany) to facilitate quantification and sample recovery. Samples were kept at −20 °C for 60 min to allow protein precipitation. After centrifugation (1200× *g*, 4 °C, 10 min), 8 mL of acidified water was added (final pH = 3.5) and the samples were subjected to solid phase extraction (SPE). The SPE cartridges (Sep-Pak^®^ Vac 6cc 500 mg/ 6 mL C18; Waters, Milford, MA, USA) were equilibrated with 6 mL methanol and then with 2 mL water prior to sample loading onto the columns. After washing with 6 mL water and an additional 6 mL *n*-hexane, LM were eluted with 6 mL methyl formate. The eluates were brought to dryness using a TurboVap LV evaporation system (Biotage, Uppsala, Sweden) and resuspended in 100 µL methanol/water (50/50, *v*/*v*) for analysis by UPLC-MS-MS. The LM were analyzed with an Acquity^™^ UPLC system (Waters, Milford, MA, USA) and a QTRAP 5500 Mass Spectrometer (ABSciex, Darmstadt, Germany), equipped with a Turbo V^™^ Source and electrospray ionization. LM were separated using an ACQUITY UPLC^®^ BEH C18 column (1.7 µm, 2.1 × 100 mm; Waters, Eschborn, Germany) at 50 °C with a flow rate of 0.3 mL/min and a mobile phase consisting of methanol/water/acetic acid of 42/58/0.01 (*v*/*v*/*v*) that was ramped to 86/14/0.01 (*v*/*v*/*v*) over 12.5 min and then to 98/2/0.01 (*v*/*v*/*v*) for 3 min [39]. The QTrap 5500 was operated in negative ionization mode using scheduled multiple reaction monitoring (MRM) coupled with information-dependent acquisition. The scheduled MRM window was 60 sec, optimized LM parameters were adopted, and the curtain gas pressure was set to 35 psi. The retention time and at least six diagnostic ions for each LM were confirmed by means of external standards (Cayman Chemical/Biomol GmbH, Hamburg, Germany). Quantification was achieved by calibration curves for each LM. Linear calibration curves were obtained for each LM and gave r2 values of 0.998 or higher (for PUFA 0.95 or higher). Additionally, the limit of detection for each targeted LM was determined [39].

### 2.3. Genome Analysis

A bioinformatics analysis using Qiagen CLC Genomics Workbench version 21.0.4 (QIAGEN Aarhus A/S, Aarhus, Denmark) of a variety of genes of interest was performed: human *LOX* genes *ALOX5* (NC_000010.11, 45374209..45446121), *ALOX12* (NC_000017.11, 6993791..7010754), *ALOX12B* (NC_000017.11, c(8072636..8087716)), ALOX15 (NC_000017.11, c(4630919..4641678)), and *ALOX15B* (NC_000017.11, 8039059..8049134), as well as *LOX* genes *PA1169* from *Pseudomonas aeruginosa* (NC_002516.2, 1267680..1269737), and *LOX1* (NC_003070.9, 20525654..20530468), *LOX2* (NC_003074.8,16525245..16529352), *LOX3* (NC_003070.9, 5977323..5981521), *LOX4* (NC_003070.9, 27308513..27312756), and *LOX5* (NC_003074.8, 7926728..7931351) from *Arabidopsis thaliana.* Protein sequences/isoforms were extracted within the Qiagen CLC software. In addition, NspLOX from *Nostoc* (Q8YK97.1) and *Bacillus megaterium* BM3 proteins (AAA87602.1) were integrated.

Therefore, 185 internally available strains of the *Bacillus subtilis* group covering *B. licheniformis*, *B. megaterium*, *B. pumilus*, *B. subtilis,* and *B. velezensis* strains were used. Draft genome sequencing of all strains was established by Illumina paired-end sequencing (2 × 150 bp). The assemblies were performed using Qiagen CLC Genomics Workbench version 12.0 with default settings (unpublished data). Genome sequences of all strains were annotated using Prokka version 1.14.5 [40]. Subsequently, protein sequences were extracted and compared to the protein of interest using the CLC Genomics Workbench version 21.0.4 BLAST interface (parameters are listed in Table 1).

Furthermore, a multiple sequence alignment of all identified BM3-like candidate proteins of *B. megaterium* strains was computed using Qiagen CLC Genomics Workbench Version 21.0.4 with default settings (gap open cost = 10, gap extension cost = 1, end gap cost = free). Based on this alignment, a phylogenetic tree was reconstructed with default settings (algorithm = Neighbor Joining, distance measure = Jukes-Cantor, bootstrap = 100 replicates).

### 2.4. Clinical Trial—Study Design & Objective

The study was performed as an open-label, single-center pilot study between September and December 2020 at the study site of the Nutritional CRO BioTeSys GmbH (Esslingen, Germany). The clinical trial was conducted as a proof-of-concept study to determine the impact of a food supplement consisting of a probiotic strain and *n*-3 PUFA on the formation and uptake of SPM and their anti-inflammatory potential over a 4-week intake phase. The primary hypothesis was that intake of the food supplement leads to a significant elevation of circulating levels of SPM and/or SPM precursors. EPA and DHA uptake and tolerability of the study product were also observed. All subjects gave their informed consent for inclusion before they participated in the study. The study protocol was reviewed by the ethics committee of Landesärztekammer Baden-Württemberg without concerns (approval number: F-2020-096). The study was registered in the German Clinical Trials Register (DRKS00023304). During an intervention period of 4 weeks, study participants received the study product. At baseline (visit 1), after 24 h (visit 2), after 1 week (visit 3), after 2 weeks (visit 4) and after 4 weeks (visit 5) of supplementation, different outcome measures were evaluated. The study was conducted in orientation to the guidelines for Good Clinical Practice (GCP) and the Declaration of Helsinki regarding the treatment of human subjects in a study.

#### 2.4.1. Study Subjects

**Figure 1:**
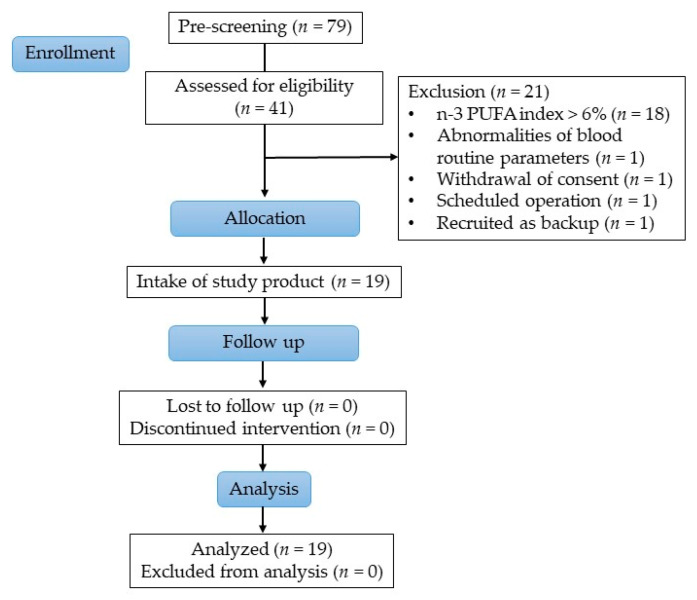
Study flow diagram.

Overall, 79 people were pre-screened for eligibility, wherefrom 41 subjects were screened to ascertain their eligibility. Healthy women between 35 and 60 years with a body mass index (BMI) between 25 and 35 kg/m^2^, non-smoker, were eligible to participate in the study. Further, subjects with low habitual consumption of fatty fish and seafood (defined as a frequency of twice per month or less) with an *n*-3 PUFA index in erythrocytes of <6.0% (determined at screening) were selected. The *n*-3 PUFA index is the sum out of EPA and DHA expressed as a percentage of total fatty acids in erythrocyte membranes. A total of 19 subjects were included in the study and all completed the study successfully, as shown in Figure 1.

The main exclusion criteria for study participation were history or presence of any severe medical disorder potentially interfering with the study (e.g., malabsorption, chronic gastrointestinal diseases, heavy depression, diabetes, acute cancers within last 3 years except basal cell carcinoma of the skin), subject under prescription for medication or taking dietary supplements possibly interfering with this study (such as *n*-3 PUFA, probiotics, anti-spasmodic, laxatives and anti-diarrheic drugs or other digestive auxiliaries, use of PPIs, bismuth salts and/or H2-antagonists, fibers etc.) within 2 weeks prior to study start or during the study, intake of antibiotics in the last 2 months, significant changes in lifestyle or medication (within last 3 months), subjects consuming food or drinks claimed as ‘probiotic’ or ‘prebiotic’ more than once weekly, consumption of more than 3 portions of fruits and vegetables (sum) per day, subjects with stool frequency of ≤ 2 stools per week or the unwillingness to abstain from fish consumption or foods/oils high in *n*-3 PUFA during the study intervention. Furthermore, subjects were asked to keep their nutrition and lifestyle habits unchanged during study participation.

#### 2.4.2. Study Product

The study product consisted of 2 billion colony forming units of *Bacillus megaterium* DSM 32963, 477 mg *n*-3 PUFA lysine salt, 180 mg hyaluronic acid, 30 mg coenzyme Q10, 20 mg lecithin from sunflower, 30 mg vitamin C, 4 mg zinc, 30 μg selenium, 20 μg biotin, 7 μg vitamin D3, 1 μg vitamin B12 (content per 2 capsules) (Evonik Operations GmbH, Darmstadt, Germany). The capsules were coated for targeted delivery of the ingredients into the large intestine. Two capsules were taken daily (one in the morning and one in the evening), unchewed with water. To allow for standardization during the study visits, subjects were instructed to take the evening capsule 12 h prior to blood sampling. Compliance of product intake was calculated from returned capsules.

#### 2.4.3. Methods for Samples and Data Collection

Blood sampling was performed at each visit after an overnight fast of at least 10 h for the determination of *n*-3 PUFA and LM that were measured in fasting plasma samples collected at baseline and end of the intervention.

Analysis of LM in plasma was performed as described in Section 2.2.

Analysis of EPA and DHA in plasma (each visit) and erythrocytes (baseline and end of intervention) was performed with gas chromatography at Omegametrix GmbH (Planegg, Germany). In brief, the fatty acid composition was analyzed using the HS-Omega-3 Index^®^ methodology as previously described [29]. Fatty acid methyl esters were generated by acid transesterification and analyzed by gas chromatography using a GC2010 Gas Chromatograph (Shimadzu, Duisburg, Germany) equipped with an SP2560, 100-m column (Supelco, Bellefonte, PA, USA) using hydrogen as carrier gas. Fatty acids were identified by comparison with a standard mixture of fatty acids characteristic of erythrocytes and plasma. A total of 26 fatty acids were identified and quantified. Results are given as a percentage of total identified fatty acids.

#### 2.4.4. Safety (Adverse Events, Concomitant Medication, and Tolerability)

Throughout the study intervention, the subjects documented any adverse events and concomitant medication in diaries. Furthermore, safety blood routine parameters and vital signs were determined. Tolerability was assessed after 1, 2, and 4 weeks of intervention.

#### 2.4.5. Statistical Analysis

The study was performed as an exploratory proof of concept study. Data obtained in this study were listed and summarized with descriptive statistics or frequency tables as appropriate. Changes versus baseline were evaluated applying paired t-test statistic or Wilcoxon signed rank test in case of non-normal distribution of data sets. Non-normality was evaluated with the Shapiro-Wilk test (*p* < 0.05). All statistical tests were performed two-sided, with a significance level of 0.05. Statistical analysis and graphs were generated using GraphPad Prism (Version 5.04, GraphPad Software, Inc., San Diego, CA, USA). Within figures, mean levels with a 95% confidence interval (CI) are depicted.

## 3. Results

### 3.1. Bacillus Megaterium Strains Harbor CYP102A1 (BM3) Gene Variants and Produce SPM and SPM Precursors from n-3 PUFA Lysine Salt

We aimed at identifying probiotic bacteria capable of self-sufficient production of SPM, i.e., without the involvement of eukaryotic host cells. Based on indications of LOX-like sequences in *Bacillaceae* [41], we conducted a bioinformatic comparison of human LOX proteins ALOX5, ALOX12, ALOX12B, ALOX15, and ALOX15B, as well as LOX proteins from *Pseudomonas aeruginosa* (PA1169) [12], *Arabidopsis* (AtLOX1-AtLOX5), and *Leuconostoc* (NspLOX) against 185 *Bacillus* genomes (from the species *Bacillus subtilis*, *Bacillus licheniformis*, *Bacillus velezensis*, *Bacillus pumilus*, and *Bacillus megaterium*). For none of the searched proteins, a hit above 70% sequence identity was observed. Only partial hits ranging from 62 to 266 amino acid lengths with percent identity values between 23.96% to 38.2% were detected and therefore LOX-like sequences appeared to be absent in the *Bacillus* sp. included in our analysis. We then searched for CYP enzyme-encoding genes previously found in the genus *Bacillus* [13]. One important member of the CYP family is CYP102A1, also termed BM3, as it has been shown to generate the SPM precursor 18-HEPE from EPA [15]. Genome sequences of a *Bacillus* sp. strain collection were screened to find candidate strains that might encode a BM3 like activity. Applying a sequence identity cutoff of at least 70%, only *Bacillus megaterium* strains were identified by this search. The strain collection contained 67 *Bacillus megaterium* strains harboring exactly one protein BLAST hit for each genome using the *B. megaterium* BM3 protein as reference. Protein sequence identity compared to the reference protein varied between 96.09% and 99.14%. Sequence identity of BLAST hits versus strains of all other *Bacillus* species ranged between 20.46% and 60.44%. Based on this clearly lower sequence identity, *B. megaterium* strains seemed to be the most promising candidates to show a CYP450BM3 like activity.

**Figure 2:**
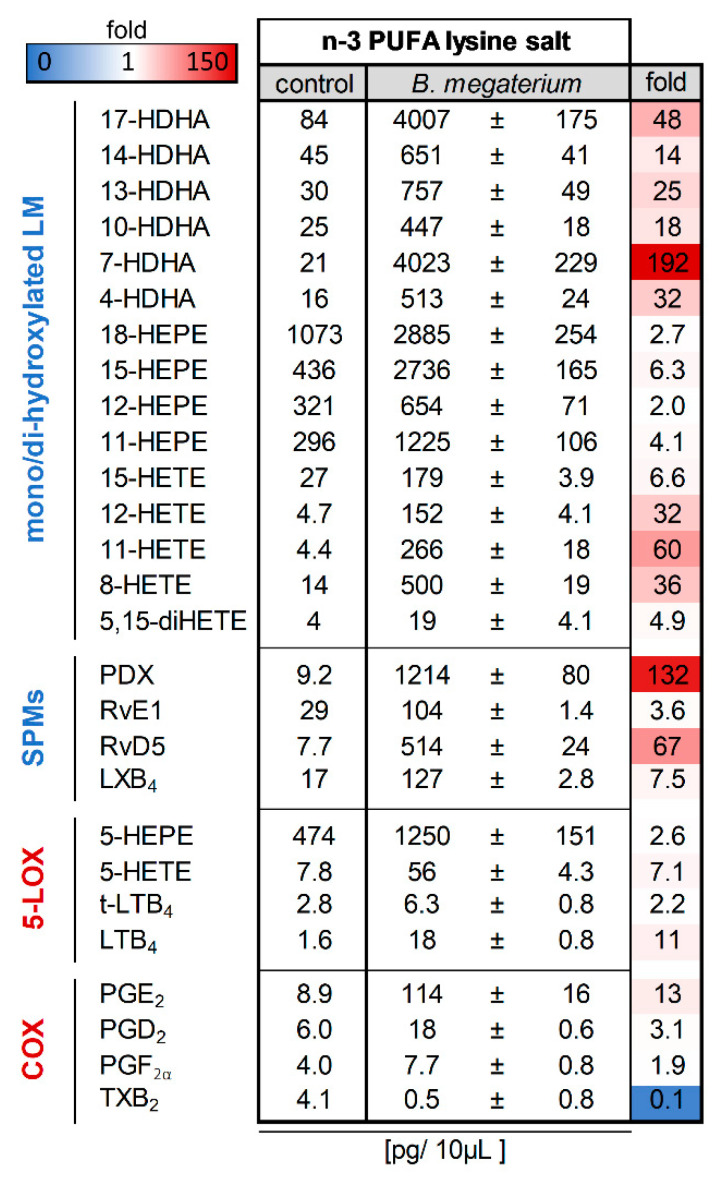
*Bacillus megaterium* DSM 32963 generates lipid mediators (LM) from *n*-3 PUFA lysine salt. Probiotic *Bacillus megaterium* DSM 32963 were cultivated with a liposomal formulation of an *n*-3 PUFA lysine salt for 17 h; for comparison, *n*-3 PUFA lysine salt was incubated in parallel without bacteria. Then LM were extracted from the supernatants of these incubations and analyzed for LM by UPLC-MS/MS. The data are given as pg/10 µL supernatant, mean ± SEM, *n* = 3 for *B. megaterium* DSM 32963-treated samples.

We then probed probiotic *Bacillus megaterium* DSM 32963 bacteria with different types of marine *n*-3 PUFA formulations followed by metabololipidomic analysis of cell culture supernatants to assess the putative formation of SPM and their precursors in addition to the previously described 18-HEPE. Pilot experiments had shown that esterified *n*-3 PUFA in the form of fish oil are poor oxygenation substrates (not shown), whereas a liposomal formulation of an *n*-3 PUFA lysine salt—wherein the fatty acids are present in their non-esterified form—gave rise to a diverse spectrum of SPM and precursors found in *Bacillus megaterium* DSM 32963 supernatants after 17 h of cultivation (Figure 2). Control reactions were performed in the absence of bacteria with *n*-3 PUFA lysine salt only, to control for spontaneous or pre-formed *n*-3 PUFA oxygenation products.

As can be seen from Figure 2, in the absence of bacteria, the incubation of the *n*-3 PUFA lysine salt led to the detection of mainly EPA-derived 18-HEPE, 15-HEPE, 12-HEPE, 11-HEPE and 5-HEPE with low amounts of DHA-derived 17-HDHA, 14-HDHA, 13-HDHA, 10-HDHA, 7-HDHA, and 4-HDHA as well as AA-derived 15-HETE, 12-HETE, 11-HETE, 8-HETE and 5-HETE. Bioactive LM typically produced by 5-LOX and COX in mammals as well as SPM were only sparsely present. Of interest, after cultivation with *Bacillus megaterium* DSM 32963, there was a strong increase in the formation of PDX, RvE1, and LXB_4_, as well as of several monohydroxylated products and SPM precursors, but also PGE_2_ and trans-LTB_4_ were strongly elevated (Figure 2).

Analysis of additional *Bacillus megaterium* strains (*n* = 47) in the identical experimental setup showed that also some other strains exhibited the capability for enhancing SPM formation from *n*-3 PUFA lysine salt with strong variations between single strains and with much lesser extent as compared to the *Bacillus megaterium* DSM 32963 strain (data not shown).

**Figure 3:**
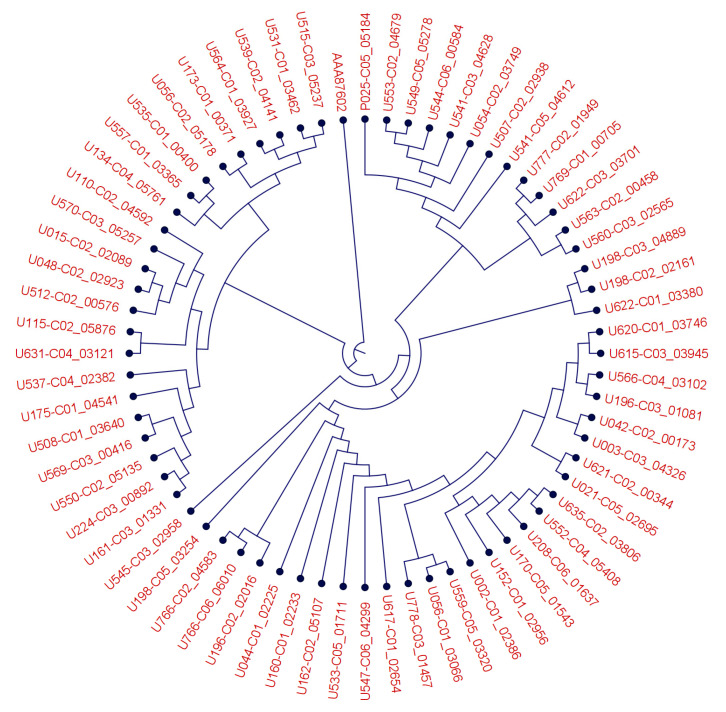
Phylogenetic tree of *B. megaterium* CYP450BM3 like proteins and the *B. megaterium* BM3 protein (AAA87602.1) demonstrating a protein sequence diversity across different strains. Root was set above AAA87602.1.

BM3 is an extremely versatile enzyme, for which numerous (engineered) point mutations have been shown to shift its substrate specificity and catalytic activity [42]. We, therefore, analyzed the BM3 protein-coding sequences of all tested strains and detected the variants shown in Figure 3. The BM3 sequence differed at up to 41 amino acid positions within the *Bacillus megaterium* consensus sequence. It thus appears likely that the BM3 sequence variation within *Bacillus megaterium* may in part explain the different experimental behavior of the strains in generating SPM and mono-oxygenated *n*-3 PUFA, though additional enzymatic activities may contribute to the observed variation as well.

Next, we prepared a synbiotic composition comprising *Bacillus megaterium* and the *n*-3 PUFA lysine salt to assess if our in vitro findings can be recapitulated in humans. As the strain *Bacillus megaterium,* DSM 32963 met all criteria for safe use in human food, laid down by the European Food Safety Authority [43], we used it for a proof-of-concept trial in humans.

### 3.2. Human Study

#### 3.2.1. Subject Characteristics

**Figure 4:**
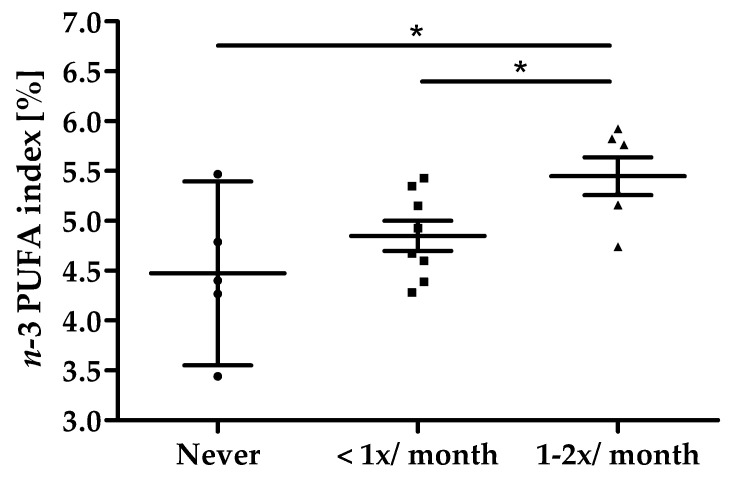
Fatty fish consumption vs. *n*-3 PUFA index at screening, scatter plot with mean ± 95% CI, * *p* < 0.05.

All 19 subjects completed the study in its entirety (Figure 1). Baseline characteristics of the study population are summarized in Table 2. All subjects reported their habitual consumption of fatty fish as twice per month or less, 5 subjects stated to never consume fatty fish. On average, a dose-dependency was seen between the frequency of fatty fish consumption and *n*-3 PUFA index at screening; however, data also show a high individual overlap between categories as shown in Figure 4. Overall, the compliance of intake of study products was high with an average compliance of 99.7% (95% CI: 97.2–102.1).

**Figure 5:**
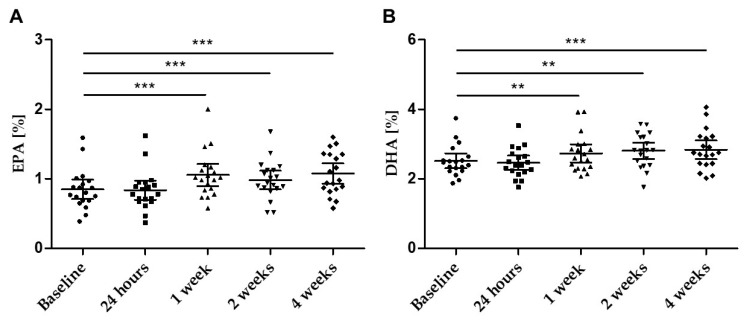
Concentration of (**A**) EPA (%) and (**B**) DHA (%) in plasma over the four weeks of supplementation at defined time points; scatter plot *n* = 19 with mean ± 95% CI; ** *p* < 0.01; *** *p* < 0.001.

The levels of EPA and DHA in fasting plasma samples increased significantly throughout the study (Table 3, Figure 5A,B). Already after 1 week of supplementation, a significant increase in both EPA and DHA was observed.

The 4-week supplementation period did not significantly affect the *n*-3 PUFA index in erythrocytes. However, the single fatty acid EPA (expressed as percentage of total fatty acids in erythrocyte membranes) increased significantly (Baseline: 0.66% (95% CI: 0.57–0.75); after 4 weeks: 0.73% (95% CI: 0.64–0.82)) in contrast to DHA (Baseline: 4.07% (95% CI: 3.82–4.32); after 4 weeks (4.10 (95% CI: 3.84–4.36)). Data reflect the composition of the study product, the EPA content was almost twice as high as the DHA content. The distribution of DHA in relation to EPA differs between plasma (3-fold) and erythrocytes (6-fold) and indicates a special enrichment/selection when incorporated in the membrane.

#### 3.2.2. SPM Levels in Plasma after a 4-Week Intervention with *B. megaterium* and *n*-3 PUFA-Lysine Salt

**Figure 6:**
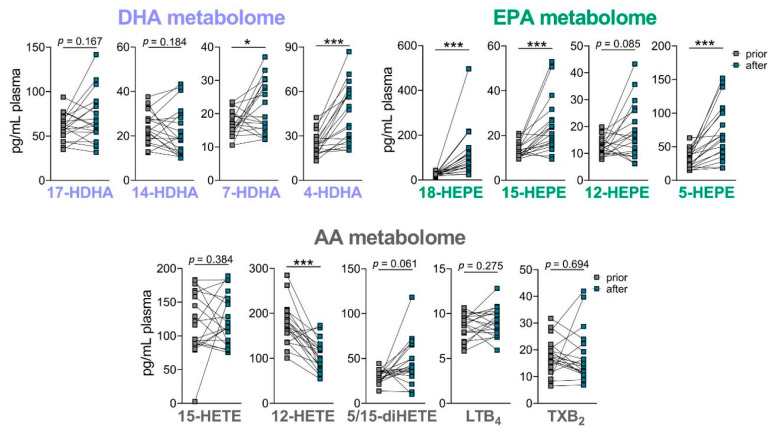
Effect of oral supplementation of *B. megaterium* and *n*-3 PUFA lysine salt on LM plasma levels. DHA-, EPA- and AA-derived LM were analyzed in the plasma of 19 volunteers prior to and after oral intake of the *B. megaterium* and *n*-3 PUFA-lysine salt. Data are given as pg/mL plasma, *n* = 19. * *p* < 0.05, *** *p* < 0.001.

We then determined the effect of the nutritional product on the PUFA-derived LM and especially on the SPM levels in the plasma of the 19 volunteers after the four-week intake phase. The basal levels of LM in the plasma of the 19 volunteers were analyzed prior to and after 4-week supplementation. As expected, the amounts of basal LM in the plasma of healthy volunteers were low, and SPM were not detected. After 4-week supplementation, there was a significant elevation of EPA-derived 18-HEPE, 15-HEPE and 5-HEPE and DHA-derived 7-HDHA and 4-HDHA (with similar positive trend for 17-HDHA), while AA-derived LTB_4_, TXB_2,_ and 15-HETE were not different at all among the detectable LM, and 12-HETE was significantly downregulated (Figure 6). These data indicate that some *n*-3 PUFA-derived SPM precursors, especially those derived from EPA, are elevated upon supplementation with the product, but not so the pro-inflammatory eicosanoids.

#### 3.2.3. Safety and Tolerability

In total, 17 adverse events were reported by nine subjects during the supplementation period. The most common reported adverse events were headache (*n* = 7) and back/joint pain (*n* = 3). None of the adverse events were classified as serious adverse events or related to the study or the intake of the investigated product. Overall, four-week supplementation was well tolerated, however, few subjects complained about the capsule size and the unpleasant odor of the product.

## 4. Discussion

Here we described the development of a novel synbiotic formulation that may serve as a self-sufficient tool for SPM generation in humans. SPM have been characterized as crucial mediators of the health-beneficial effects of their precursors, *n*-3 PUFA and *n*-6 PUFA, with respect to the resolution of chronic inflammatory conditions [44,45]. Although animal disease models of e.g., colitis [24] have shown effective treatment effects by SPM, clinical trials with *n*-3 PUFA have yielded inconclusive or null results, especially for patients with IBD, asthma, and traits of the metabolic syndrome [2,33,46,47]. Obviously, the progression from *n*-3 PUFA intervention towards SPM formation/action and inflammation resolution is affected by numerous confounders, which may partly explain disappointing/mixed outcomes as summarized in recent meta-analyses of *n*-3 PUFA trials. Important confounders in these trials are baseline *n*-3 PUFA status, the chemical identity and stability of the study product, and individual modulators of pharmacokinetics such as diet, medication, age, and genetics.

Importantly, the biosynthesis of SPM and subsequent signaling events triggered by them seem to be dysregulated under pathologic conditions, as has been exemplified for severe asthma [34], IBD [11,36,37,48], metabolic syndrome [33], cystic fibrosis [38], and periodontitis [49]. Targeting the PUFA-to-SPM conversion efficacy may therefore be a lever towards improving health outcomes and led us to conceive an SPM-generating formulation that functions self-sufficiently, i.e., not depending on the host’s biochemical potential for de-novo biosynthesis. Of note, the in-situ production of SPM has advantages over the direct delivery of pre-synthesized SPM (in the form of e.g., pharmaceutical formulations or enriched fish oils), for example by enabling a sustained delivery and outlined in more detail in the introduction section.

An ectopically expressed BM3 protein has been shown to convert EPA to 18-HEPE [15]. Based on this finding, we searched the genomes of aerotolerant, probiotic bacteria for BM3 sequences and found them exclusively in the species *Bacillus megaterium*. We observed that, in addition to 18-HEPE, a broad spectrum of mono- and dihydroxylated lipid mediators and SPM was produced from supplemental *n*-3 PUFA by *Bacillus megaterium* strains in vitro. Whether this diversity results from BM3 activity alone or involves other enzymes as well remains to be established. The BM3 enzyme is, however, extremely versatile, as has been summarized by Thistlethwaite et al. [42], and it is conceivable that it can perform subsequent oxygenations at multiple positions in the substrates EPA, DHA, and AA. Single amino acid changes are reportedly sufficient to shift the substrate specificity of BM3 [42], and this may explain the diversity of SPM profiles generated by the 48 *Bacillus megaterium* strains we assessed here (not shown), and for which we found the BM3 protein sequences to vary at up to 41 positions (homology to AAA87602 = 96.09–99.14%; equivalent to 9–41 amino acid changes within 1049 amino acids in total). As to the possible involvement of other PUFA-oxygenating enzymes we searched *Bacillus megaterium* for the presence of LOX and COX genes (from e.g., *Homo sapiens*, *Leuconostoc*, *Pseudomonas aeruginosa*, *Anabena*, *Nostoc*), but found only hits of less than 60 bp and thus concluded their absence in all of the 48 strains we assessed.

Isolated *Bacillus megaterium* strains were unable to convert esterified PUFA into SPM. Although a complex microbiota (as in the gastrointestinal tract) may provide the lipase activities required to release EPA and DHA as free fatty acids and thus makes them available to *Bacillus megaterium*, we here developed a synbiotic nutritional formulation based on the same *n*-3 PUFA lysine salt that we successfully applied in vitro and used this for the in vivo trial. Using this salt also solved the technical challenge of maintaining the viability of dried bacteria in combination with (liquid) fatty acids, as we observed excellent stability of both the PUFA and *Bacillus megaterium* DSM 32963 bacteria for more than two years. To our knowledge, the development and application of a formulation combining non-esterified PUFA with microorganisms have not been described before. Our in vivo trial was conducted as a proof-of-concept study for the elevation of circulating SPM and precursors as the primary outcome. The four-week intervention with the study product led to significantly increased levels of mono-hydroxylated LM derived from EPA, DHA, and AA, which were also formed *in vitro*. In contrast, product spectra observed under in vivo versus in vitro conditions were divergent regarding di- and tri-hydroxylated *n*-3/*n*-6 PUFA (SPM). This divergence is most likely the result of different pharmacokinetic behavior; in fact, the uptake of oxygenated PUFA from the gastrointestinal tract has not been described in detail. We may speculate that the oxygenation level of EPA, DHA, and AA affects their (intraluminal) half-life and their absorption. It will be worthwhile to study the formation and fate of oxygenated PUFA in a complex microbiota. Regarding the availability of oxygen, it is plausible that *Bacillus megaterium* DSM 32963-derived BM3 did perform oxygenations also in the gastrointestinal tract, as previous studies have shown that this environment is not strictly anaerobic, even in the colon [50,51,52,53]. Although the intraluminal oxygen level is low, it increases (from 1 to 40 mmHg) towards the vascularized mucosa, indicating that oxygen diffuses from intestinal tissue into the lumen [50]. This oxygen gradient results in an enrichment of mucosa-associated microbiota with aerotolerant and aerobic taxa such as *Proteobacteria* [50,51], indicating that the *Bacillus megaterium* DSM 32963 applied here may have integrated into this niche and delivered significant amounts of LM—at higher concentrations as found in plasma.

Intraluminally produced SPM and precursors thereof are of relevance for gastrointestinal physiology in general [54] and for chronic intestinal diseases, namely IBD and colorectal cancer, in particular. An impaired SPM biosynthesis has been reported for IBD [11,55], which may explain the overall lack of benefit from *n*-3 PUFA supplementation for IBD patients [46,47]. On the other hand, 15-HEPE [56], RvE1 [24], 17-HDHA, RvD_1_, RvD_2_ [57], MaR_1_ [58], and LXA_4_ [59] are protective in murine models of IBD, whereas 15-HETE and 18-HEPE have proposed roles in supporting intestinal barrier resistance and epithelial healing in patients with Crohn’s disease [36]. As we observed significant production of several of these LM by our synbiotic combination both in vitro and in humans, it is meaningful to test its therapeutic potential in IBD. Moreover, delineation of the functions of specific SPM in gastrointestinal diseases may pave the way towards targeted synbiotic treatment strategies based on *Bacillus megaterium* strains with specific BM3-metabotypes. Our synbiotic approach may also be used in other applications where a local in situ production of SPM is beneficial, e.g., conditions on the skin or in the oral cavity, using suitable carriers or matrices.

A limitation of our human trial is the small sample size, the uncontrolled study design, and that the study product also contained additional ingredients (vitamins, zinc, selenium, hyaluronic acid, coenzyme Q10, lecithin, biotin), whose involvement in endogenous production of SPM is unlikely but cannot be excluded. The biochemical actions of our synbiotic formulation need to be elucidated further, especially within a complex microbiota. Clearly, additional and larger studies are warranted to confirm and expand our findings presented here.

## 5. Conclusions

We identified that bacteria of the species *Bacillus megaterium* can produce a broad range of SPM and precursors thereof from an *n*-3 PUFA lysine salt preparation. A nutritional synbiotic combination of *Bacillus megaterium* DSM 32963 increases plasma levels of EPA-, DHA-, and AA-derived mono-hydroxylated LM. Overall, this study describes a novel route towards increasing SPM levels in vivo based on synbiotic formulations comprising bacteria of the species *Bacillus megaterium* and a suitable (= stable and bioavailable) *n*-3 PUFA source. This route acts self-sufficiently and can be targeted towards e.g., the intestine as well as the formation of beneficial SPM profiles; it may therefore overcome the limitations of conventional *n*-3 PUFA supplementations and provide a basis for novel strategies to resolve inflammatory conditions and treat associated diseases. This implies a possible relevance of *Bacillus megaterium*/*n*-3 PUFA lysine salt combinations in the treatment of IBD as well as extraintestinal inflammatory diseases such as arthritis.

## 6. Patent Applications

PCT/EP2019/082924.

PCT/EP2019/082935.

PCT/EP2019/082919.

## Figures and Tables

**Table 1:** BLAST (Basic Local Alignment Search Tool) parameters for intrinsic occurrence analysis.

Program	CLC Genomics Genomics Workbench 21.0.4 BLASTp
Blast version	2.9.0+
Scoring Matrix	BLOSUM62 (BLOcks SUbstitution Matrix) (Existence 11, Extension 1)
Minimum query coverage	60%
Minimum identity	70%
Word size	3
Maximum number of hits versus a database	1500

**Table 2:** Demographic data and baseline characteristics (*n* = 19, at screening). Data are shown as means (95% CI).

Variables	Study Population
Age (years)	51.0 (47.7–54.3)
BMI (kg/m^2^)	28.3 (27.1–29.5)
Cholesterol (mg/dL)	209.5 (189.6–229.4)
LDL-cholesterol (mg/dL)	135.1 (116.4–153.8)
GPT (U/L)	27.7 (20.9–34.4)
GOT (U/L)	20.8 (17.9–23.7)
Systolic blood pressure	131.2 (123.2–139.2)
Diastolic blood pressure	87.3 (83.4–91.3)
*n*-3 PUFA index in erythrocytes	4.9 (4.6–5.2)

BMI: body mass index; LDL: low-density lipoprotein; GPT: glutamate pyruvic transaminase; GOT: glutamic oxaloacetic transaminase; PUFA: polyunsaturated fatty acids.

**Table 3:** EPA and DHA in fasting plasma samples (*n* = 19).

Variables	Baseline	24 h	1 Week	2 Weeks	4 Weeks
	Mean (95% CI)	Mean (95% CI)	Mean (95% CI)	Mean (95% CI)	Mean (95% CI)
EPA [%]	0.85 (0.71–0.99)	0.84 (0.70–0.97)	1.06 (0.90–1.21) *p* = 0.0002	0.98 (0.85–1.12) *p* = 0.0008	1.08 (0.93–1.22) *p* = 0.0006
DHA [%]	2.52 (2.30–2.73)	2.46 (2.26–2-67)	2.73 (2.47–2.99) *p* = 0.0055	2.81 (2.57–3.04) *p* = 0.0029	2.84 (2.57–3.11) *p* = 0.0006

## Data Availability

The data presented in this study are available on request from the corresponding author. The data are not publicly available due to patenting processes.
